# Fe_3_O_4_/Mulberry Stem Biochar as a Potential Amendment for Highly Arsenic-Contaminated Paddy Soil Remediation

**DOI:** 10.3390/toxics12110765

**Published:** 2024-10-22

**Authors:** Ziling Tang, Meina Liang, Yanmei Ding, Chongmin Liu, Qing Zhang, Dunqiu Wang, Xuehong Zhang

**Affiliations:** 1 College of Environmental Science and Engineering, Guilin University of Technology, Guilin 541004, China; 2 Guangxi Key Laboratory of Environmental Pollution Control Theory and Technology, Guilin University of Technology, Guilin 541006, China; 3 Guangxi Collaborative Innovation Center for Water Pollution Control and Water Safety in Karst Area, Guilin University of Technology, Guilin 541006, China

**Keywords:** arsenic, magnetic biochar, paddy field, soil remediation

## Abstract

Magnetite-loaded biochar has recently received attention owing to its ability to remove arsenic from contaminated soil. In this study, mulberry stem biochar (MBC) and Fe_3_O_4_-loaded mulberry stem biochar (Fe_3_O_4_@MBC) were produced and used in a 100-day incubation experiment to investigate their performance in the stabilization of arsenic in paddy soil severely polluted by the As (237.68 mg·kg^−1^) mechanism. Incubation experiments showed that Fe_3_O_4_@MBC was more effective in immobilizing As after incubation for 100 days. Moreover, adding Fe_3_O_4_@MBC facilitated the transformation of exchangeable heavy metals into organic-bound and residual forms, thereby reducing As available concentrations, mobility, and bioavailability in the soil, and elevating slightly the soil pH and dissolved organic carbon (DOC). The concentration of TCLP-extractable As (As_TCLP_) in contaminated soil was reduced from 93.85 to 7.64 μg·L^−1^ within 10 d, below the safety limit for drinking water set by the World Health Organization (WHO). The characterization results of Fe_3_O_4_@MBC after incubation indicated that the mechanisms for As passivation are linked to redox reactions, complexation, electrostatic attraction, surface adsorption, and coprecipitation. Conclusively, Fe_3_O_4_@MBC is a promising amendment in highly As-contaminated soil and provides a theoretical reference in such polluted paddy soil remediation.

## 1. Introduction

Arsenic is a poisonous and common element, and As contamination issues have been a social concern for decades [1,2,3]. According to the Report on the National Soil Contamination Survey in China, 19.4% of China’s agricultural soils (equivalent to approximately 26 million ha) are contaminated, mainly with heavy metals and metalloids (HMs) [4]. Moreover, 2.7% of the investigated sites covering over 65% of China’s land area exceeded the regulatory limit of As, which ranked third among the eight monitored inorganic contaminants [5]. Arsenic is a persistent environmental contaminants that is difficult to degrade, and long-term or high-dose short-term As exposure increases the risk of cancer in numerous organs and can result in a number of disorders connected to the circulatory and reproductive systems [6]. Its long-term accumulation can alter soil properties, impact crop quality, and ultimately pose serious potential health problems for humans by entering the body through food chains like “soil–plant–(animal)–human” [7]. Thus, it is urgent to control soil arsenic pollution and reduce the bioavailability of arsenic in contaminated soil.

The techniques used to remediate As in soil can be broadly categorized into physical, chemical, and biological methods [8,9]. The large-scale application of the above remediation techniques is often limited by incomplete techniques, theoretical shortage or high cost. Considering factors such as labor cost, time cost, technological maturity, and applicability, the most common approach for remediating As in soil involves the application of passivators [8,10]. Various amendments, such as lime, fly ash, red mud, green waste, compost, and various minerals have been applied for As immobilization in contaminated soil [11]. Flouring researchers have devoted themselves to finding more functional materials in terms of cost, performance, efficiency and availability. Nevertheless, the stabilization mechanisms of soil amendments can vary, and the migration of heavy metals is influenced by numerous factors. The pattern of change in reactive arsenic concentration in soil solution is not clear.

Numerous studies have proven that biochar application can exhibit great potential for HM-contaminated soil remediation because of its high negative charge density, highly porous structure, large surface area, and numerous functional groups, such as hydroxyl, and carboxyl groups [12,13,14], and its incorporation into soils can provide a beneficial long-term carbon sink [13]. However, pristine biochar has limited capability in remediating As-contaminated soil unless it is modified or functionalized to improve its capability [15]. In comparison, Fe oxide-based materials have demonstrated a considerable remediation ability for As in batch, soil incubation and pot experiments [16,17,18]. This is because Fe oxides have less effect on the physicochemical properties of soil and can effectively prevent the migration of arsenic in soil and reduce As bioavailability. The main remediation mechanism of arsenic-contaminated soil by Fe oxide is to fix arsenic on Fe oxides by precipitation, complexation, and adsorption to reduce the mobility and bioavailability of arsenic [16]. Thus, it was proposed that combining Fe oxide with biochar would create a magnetic biochar material to immobilize the contamination of As.

Previous literature studies have shown that the amount of arsenic in rice seeds can be considerably decreased by applying and incorporating iron oxide and magnetic iron oxide/biochar [18,19,20]. The adsorption and immobilization of arsenic by biochar was enhanced by altering its surface characteristics with iron oxide [21]. Because of its magnetic properties, the dry magnetic separation method can be used to isolate magnetic biochar from the soil, and the resultant product can collect 25% of the total arsenic present in the soil [21,22]. A combined immobilization and magnetic retrieval approach may have the potential not only for As immobilization but also for the permanent removal of As from soils [14,23]. However, little is known about the effect and related mechanism of Fe oxide biochar on As-contaminated soil, such as soil physicochemical (e.g., pH, organic matter), which play a crucial role in interaction mechanisms between amendments and arsenic in any specific treatment [24,25]. The total As contents in amended soils remain unchanged, and the effectiveness of immobilization requires long-term monitoring [26,27]. Given the above, it is essential to create remediation materials appropriate for soils with elevated levels of arsenic contamination and to clarify how they work. 

The primary objectives of this study were (1) to investigate using mulberry as basic material to prepare magnetic carbon-based composites with higher pH_zpc_ and less effect on soil pH; (2) to examine the forms of As-contaminated paddy soil arsenic and soil physical and chemical properties after different level treatments; (3) to elucidate the mechanism of As passivation by Fe_3_O_4_@MBC in As-contaminated paddy soil; and (4) to provide a lower-cost, effective and readily available in situ remediation technology in the field of highly As-contaminated farmland soil.

## 2. Materials and Methods

### 2.1. Materials

Test soil was collected from the top layer (0–20 cm) of paddy field located in Dachang, Nandan, Hechi, Guangxi province, China (107°59′96″ E, 4°85′61″ W). The soil was naturally air-dried, ground and mixed evenly, passed through a 0.15 mm sieve, and sealed for later use. The concentration of total As was 237.68 mg·kg^−1^, with a Nemerow comprehensive contaminate index value of 6.35 (high level of contamination). The basic physical and chemical properties of soil are listed in Table S1, and the arsenic form in test soil is listed in Table S2. The mulberry stems used in this study were collected from local farms. The experiment reagents are written in the Supplementary Materials.

### 2.2. Experimental Methods

#### 2.2.1. Preparation of MBC and Fe_3_O_4_@MBC

**Figure 1:**
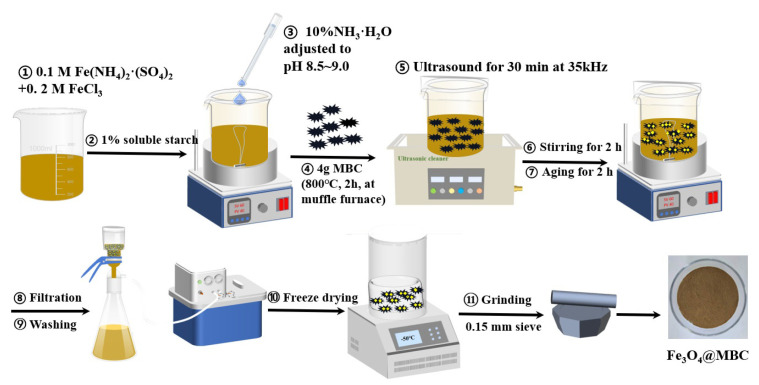
A schematic diagram showing the preparation of Fe_3_O_4_@MBC composites.

MBC and Fe_3_O_4_@MBC were synthesized according to our previous research method [10] as follows: 400 mL of a mixed solution of 0.1 M Fe^2+^ and 0.2 M Fe^3+^ (prepared on the spot) was added to a 1000 mL beaker followed by magnetic stirring at 240 rpm for 5 min; the mixed solution was titrated with 10% ammonia solution to pH 8.5–9.0 under magnetic agitation at 240 rpm; then 4 g of MBC (mulberry stem powder was placed in a muffle furnace and carbonized at 800 °C for 2 h at a heating rate of 5 °C·min^−1^) was merged into the mixed solution. The resulting mixture was performed ultrasonically at 35 kHz for 30 min, stirred magnetically at 70 °C for 2 h, and then aged for 4 h. Subsequently, the obtained product was cooled and filtered, washed with ultra-pure water to washing liquid pH around 7.0, and then washed twice with anhydrous ethanol and centrifuged. The resulting filter cake was freeze-dried at −50 °C for 48 to 72 h. In the end, these materials were ground to pass a 0.15 mm sieve and stored. The preparation process is illustrated in Figure 1.

#### 2.2.2. Soil Incubation Experiments

The soil incubation experiments were performed with soil (50g, ≤0.15 mm) in a 100 mL wide-mouth polyethylene plastic bottle. Dosages of 1–7% (*w/w*) MBC and Fe_3_O_4_@MBC were separately mixed into soils at the same time. A perforated plastic cap was used to seal the mouth of the bottle and ensure uniform water evaporation. Three replicates were performed in parallel for each treatment and the control check group (CK). Holding the temperature at 25 ± 2 °C and using the weight method, the soil moisture capacity was maintained at approximately 70% of the field moisture capacity. Soil moisture was adjusted every 4 d. Soil samples were collected after incubation for 1, 10, 20, 30, 50, 70, and 100 d. Finally, the collected soil samples were dried and clustered to pass through a 0.15 mm sieve at the appointed time. They were used to analyze the properties of soil and to conduct the soil arsenic speciation analysis and soil toxicity leaching experiments. 

#### 2.2.3. Characterization and Determination of Material and Soil Samples

The carbon (C), hydrogen (H), nitrogen (N), and sulfur (S) concentrations in the biochars were determined using an elemental analyzer (Perkin Elmer EA2400II, Waltham, MA, USA). Brunauer Emmett Teller (BET) surface area measurements, Zeta potential and scanning electron microscopy (SEM) of these raw materials were performed as previously described [10]. After incubation, the Fe_3_O_4_@MBC particles after incubation were recovered via magnetic separation from the 7% Fe_3_O_4_@MBC amended treatment sample taken after 100 d of incubation. After incubation, Fe_3_O_4_@MBC and Fe_3_O_4_@MBC were analyzed by X-ray diffraction (XRD, Bruker-axs D8ADVANCE, Germany, Karlsruhe), Fourier-transform infrared spectroscopy (FTIR, NICOLET6700, Japan), and X-ray photoelectron spectroscopy (XPS, Thermo Scientific K-Alpha, Waltham, MA, USA) were used to clarify the fundamental properties and remediation mechanisms of Fe_3_O_4_@MBC. The binding energy of C 1s (284.8 eV) was used as the reference peak for charge correction.

The soil pH was measured using a pH meter (E-201-C, China, Shanghai) at a soil-to-water ratio of 1:2.5(HJ 962-2018, China, Beijing). Soil EC was measured using a conductivity meter (DDS-801, China, Shanghai) at a soil-to-water ratio of 1:5. The soil was shaken at 20 ± 1 °C for 30 min, then centrifuge and the supernatant was collected for measurement (HJ 802-2016, China, Beijing). The soil DOC content was tested using a TOC-1020A organic carbon analyzer (Multi N/C 3100, Germany, Analytik Jena). To determine the bound forms of As in the soil before and after remediation, a selective sequential extraction approach was used, according to the European Community Bureau of Reference (BCR) gradual separation technique [28]. Quantitative analysis of total, available, water-soluble, and bound As in the soil was performed using atomic fluorescence spectroscopy (AFS, GB/T 22105.2-2008, China, Beijing). Soil labile As was extracted using Toxicity Characteristic Leaching Procedure (TCLP) analysis [16,29]. The As concentrations in the extraction fluid were filtered through 0.22 μm nylon membrane filters and analyzed via AFS.

#### 2.2.4. Quality Control and Statistical Analyses

Quality control of the As measurement was conducted using standard and reagent blanks for every ten samples. Certified standard reference materials for total As assessment in the soil (GBW 07401) sample were obtained from the China Standard Materials Research Center. The recovery rate of the total As was 91.44–118.70%. All experimental data were processed using Origin 2023 and Excel 2016 software, and a one-way ANOVA test was performed by SPSS 23.0. Principal component analysis (PCA) and Pearson’s correlation analysis were performed using Origin 2023 to confirm the relationship among different soil parameters and treatment.

## 3. Results

### 3.1. Impacts of Fe_3_O_4_@MBC on Soil Properties 

#### 3.1.1. pH 

Generally, pH is an essential parameter affecting the remediation efficiency of arsenic pollution [19,30]. After 1–100 d incubation experiments, the pH analysis results of adding 1–7% MBC and Fe_3_O_4_@MBC into the soil are presented in Figure S1. The dosages of MBC and Fe_3_O_4_@MBC significantly (*p* < 0.05) increased the soil pH. Compared with CK (pH = 6.18), adding 1–7% Fe_3_O_4_@MBC increased the soil pH by 0.03 to 0.68 units. Adding MBC raised the pH by 0.32 to 1.70 units and showed the greatest increase, from 6.18 to 7.88 (day 70, Figure S1d). The pH enhancement capacity of the two materials followed the order MBC > Fe_3_O_4_@MBC. This is because biochar is an alkaline substance, which can slowly release alkali and alkaline earth minerals and bind negatively charged functional groups with soil H^+^ ions on its surfaces [17,30]. Previous research findings also suggested that moist-incubating conditions promote the gradual release of basic ions (e.g., carbonates, phosphates, and silicates) on the biochar surface into the soil [31,32], which leads to the consumption of protons in soil particle solution, thereby increasing soil pH during the initial incubation stage (1–30 d). After 30 d, the pH values of all soil samples remained largely steady. Comparatively, the Fe_3_O_4_@MBC had a slight change by elevating soil pH in the entire cultivation cycle, which avoided the shortcomings of high soil pH caused by the addition of MBC and low soil pH by Fe_3_O_4_ [21]. Moreover, calcium-based magnetic rice straw biochar-loaded iron also significantly affect the pH of paddy soils [28], similar to our result. This indicates that single MBC and Fe_3_O_4_@MBC may have different chemical properties, especially their pH values. Fe_3_O_4_@MBC is not simply a direct combination of Fe_3_O_4_ and biochar alone but can combine the advantages of both.

#### 3.1.2. EC 

Soil electrical conductivity (EC) reflects the content of total salt ions (cations and anions) in soil leachate and has a good correlation with the bioavailability of HMs in soil [33,34]. Usually, an increase in soil EC is accompanied by an increase in the content of nutrients, such as Ca, K, Mg, and Na, which compete with HMs for adsorption sites, thereby accelerating the mobility of HMs [35]. The effects of adding 1–7% MBC and Fe_3_O_4_@MBC on soil EC values after 1–100 d of incubation are summarized in Figure S2. The soil EC values significantly decreased (*p* < 0.05) with the increase in the Fe_3_O_4_@MBC’s dosage (3–7%). This phenomenon can be attributed to the application of Fe_3_O_4_@MBC that increased soil pH. As shown in Figure S5, when the soil pH > pH_zpc_ (Fe_3_O_4_@MBC) = 3.8 (the point of zero potential (pHzpc) is the pH value corresponding to a Zeta potential of 0 mv), following deprotonation of oxygen-containing functional groups, the surface of Fe_3_O_4_@MBC was negatively charged, which was favorable for promoting electrostatic adsorption and precipitation of soluble cations (e.g., K^+^, Ca^2+^, Na^+^, Mg^2+^, etc.) in the soil. Moreover, biochar has a rough surface, abundant pores and high-temperature pyrolysis preparation conditions, making it highly capable of adsorbing soil moisture and salt [18,31]. The EC of CK on the first day was 144.9 μS·cm^−1^ (Figure S2a), which indicates the low content of soluble ions in the paddy soil (the salinity permissible range of <0.3 dS·m^−1^ [24]). With the increase in the MBC addition ratio and cultivation time, the EC of the CK sample gradually increased to 301.50 μS·cm^−1^, whereas the EC values after adding 7% MBC treatment soil and 7% Fe_3_O_4_@MBC treatment soil were 246.13 and 191.57 μS·cm^−1^ on 100 d of incubation, respectively. A study reported that 3% ferrihydrite-supported animal-derived biochar can reduce soil EC because it slows down the dissolution of silicates and carbonates [32]. Both MBC and Fe_3_O_4_@MBC are effective in reducing soil EC values, but Fe_3_O_4_@MBC performs better. From another perspective, the Fe_3_O_4_@MBC amendments have the potential to effectively immobilize soluble ions effectively, especially HMs.

#### 3.1.3. DOC 

Soil DOC is most related to the organic binding state of HMs and can serve as an electron shuttle to facilitate the transformation of Fe oxides and the redistribution of As [36,37]. One or more of the following mechanisms could be at play: (1) biochar encourages DOC’s oxidative breakdown, which results in the loss of electrons, while As (V)/Fe (III), the electron acceptor, experiences a reduction reaction upon receiving electrons, (2) biochar encourages DOC’s humification process in the liquid phase layer, wherein As/Fe reducing bacteria convert more organic matter into humus, which serves as an electron mediator to control the reduction of As (V)/Fe (III) [21,38]. 

After the addition of 1–7% MBC and Fe_3_O_4_@MBC to As-contaminated paddy soil, the soil DOC content rapidly increased within 1–10 d of incubation, but it sharply decreased within 10–20 d incubation (Figure S3). Compared with the CK group (75.17 mg·kg^1^), the content of DOC in the soil after the addition of MBC and Fe_3_O_4_@MBC increased to 80–120 mg·kg^1^ on the 10th day. The DOC contents in the different treatments were ranked as CK < Fe_3_O_4_@MBC < MBC. The higher dosage of pristine MBC treatment significantly enhanced the concentration of DOC in the soil. This phenomenon can be explained as follows: applying biochar could increase the DOC content because of the direct release of DOC from biochar itself and the facilitation of increased soil DOC [16,34]. Previous studies have also shown that increased DOC improves As adsorption on nano-mineral surfaces in acidic settings, where it binds to arsenate to produce organic As small-molecule complexes; increased DOC in alkaline environments competes with As for adsorption sites on the surface of soil minerals, which improves the solubility and mobility of arsenic [38,39,40]. We concluded that a decrease in soil arsenic bioavailability is hindered by an increase in soil DOC content. MBC treatment significantly increased the DOC content compared with the Fe_3_O_4_@MBC treatment at the same dosage. Considering that the total carbon content of Fe_3_O_4_@MBC was lower than that of MBC at the same dosage (Table S3) and combined with the DOC results of treatment samples, we speculated that Fe_3_O_4_@MBC exhibited a better carbon sequestration effect than MBC. The ferrihydrite component of 3% FAB can form a complex or precipitate with DOC in the soil during the whole incubation period (150 d) Bamboo biochar and nZVI@BC (1:3) applications increased paddy soil’s DOM content [35,41]. This finding is consistent with our results. Fe_3_O_4_@MBC may have considerable capacity to reduce the mobility of DOC-bound HMs.

### 3.2. Impacts of Fe_3_O_4_@MBC on Arsenic Availability and Fractions in Soil 

#### 3.2.1. Available Arsenic

**Figure 2:**
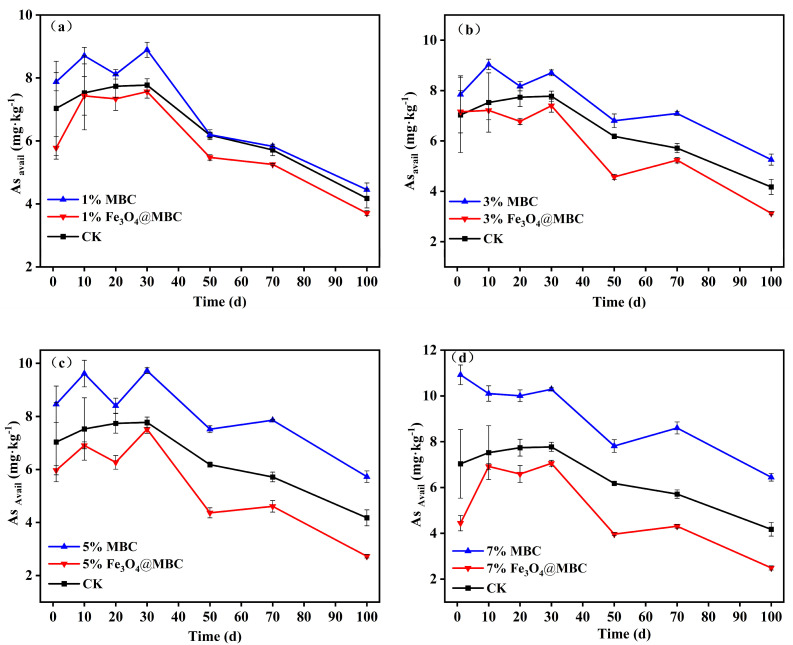
(**a**–**d**). The effects of soil As_avail_ content after adding 1%, 3%, 5%, 7% MBC and Fe_3_O_4_@MBC.

Non-specifically bound As and specifically bound As are considered to have strong bioavailability and migration ability [37]. A NaHCO_3_ solution was utilized to extract them. The majority of non-specifically bound As is on the surface of soil particles, where it can enter the soil solution by ion exchange; specifically bound As in soil forms an interior layer of adsorption mostly on the surface of iron and manganese oxides in soil particles. The dynamic changes of 1–7% MBC and Fe_3_O_4_@MBC on available arsenic (As_avail_) in the soil are indicated in Figure 2. Soil As_avail_ was positively correlated (*p* < 0.01) with pH (Figure S1). As demonstrated by Equation (1), arsenic typically occurs in soil as an oxygenated anion and is subject to valence shifts between As(III) and As(V). The release of As from soil adsorption sites is facilitated by an increase in OH^−^ in high pH environments [42]:

H_(3−n)_AsO_4_^n−^ + Soil-(OH)_n_ ⇔ Soil-H_(3−n)_AsO_4_ + nOH^−^ (1)

The concentration of As_avail_ in the CK group was 7.03 mg·kg^1^ on the first day of soil incubation, and the immobilization rates of available As in all treatments changed significantly (Figure 2). For the CK group, all treatments’ As_avail_ concentrations increased from 1 d to 30 d of soil incubation and subsequently steadily decreased from 30 d to 100 d of soil incubation; on 100 d incubation, the As_avail_ of adding 7% Fe_3_O_4_@MBC treatment soil was 2.49 mg·kg^1^, decreasing by 64.58% (Figure 2d). Further, it was dosage-dependent for the As passivation rate as the addition of Fe_3_O_4_@MBC. The trend in the As_avail_ of treatment soil change in adding MBC was the same as in adding Fe_3_O_4_@MBC at the same dosage, yet the effect of Fe_3_O_4_@MBC on the As_avail_ of soil was less than that of MBC. This may be because the higher pH values were more favorable for the release of labile As in soils [18,26]. These results indicate that Fe_3_O_4_@MBC has a remarkable reducing effect on available arsenic in the soil compared with MBC. The higher the addition ratio of Fe_3_O_4_@MBC, the stronger the passivation effect on the available arsenic in the soil [43].

#### 3.2.2. Water-Soluble Arsenic

**Figure 3:**
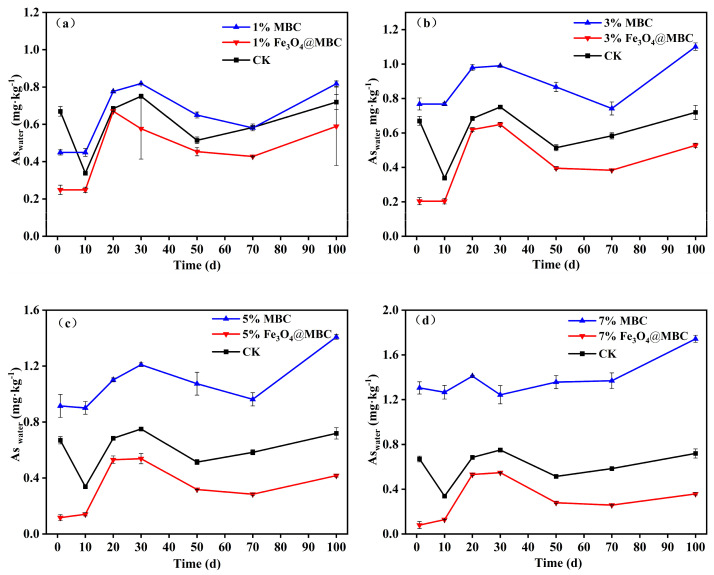
(**a**–**d**). Effect of MBC and Fe_3_O_4_@MBC treatments (1%, 3%, 5%, 7%) on soil As_water_ concentration at different incubation times.

According to previous studies, the amount of As_water_ and non-specifically bound As in contaminated soils correlates well with the amount of arsenic in plants, and most of this arsenic can be absorbed by plants [40]. AsO_4_^3−^ and AsO_3_^3−^ are the two primary forms of water-soluble arsenic (As_water_), which is typically found in soil solutions [13,44]. Therefore, As_water_ is the primary assessment indicator of environmental risk. As shown in Figure 3, for the soil collected at different times, increasing the addition rate of Fe_3_O_4_@MBC significantly decreased the soil As_water_ concentration compared with the control, which was attributed to the transformation to the residual fraction over time. However, MBC greatly increased the soil As_water_ concentration for all treatments. In the doses of 1%, 3%, 5%, and 7% Fe_3_O_4_@MBC treatments, the As_water_’s immobilization efficiency was found to increase from 19.31% to 26.92% (Figure 3a), from 30.59% to 40.16% (Figure 3b), from 58.70% to 82.57% (Figure 3c), and from 62.30% to 88.59% (Figure 3d), in comparison with the control (1–10 d). Then, the concentration of As_water_ kept the growth trend as the incubation days increase continuously (10–30 d). Eventually, it kept slight changes during the 50th through the 100th day of incubation time. This implied that the addition of Fe_3_O_4_@MBC played a positive role in As immobilization in the soil, and Fe minerals control the status of soil As whatever the various ratio treatments [21,34]. Consistent with our analysis above, a single MBC can activate As, so it is better to use it as a substrate or carrier to combine with iron oxides to achieve As passivation [37,45]. The cost of a single iron oxide material (CNY 4800 ha^−1^) is much higher than that of iron-based modified biochar materials (CNY 2336 ha^−1^) [10,31]. Overall, taking account of the economic benefits and environmental factors, it is necessary to modify MBC to stabilize As and other anionic metals in contaminated soils.

#### 3.2.3. Bound Arsenic

This study used the BCR extraction method to analyze soil-bound As, which can be divided into four types [40] as follows: (1) mild acid-soluble fraction (F1), (2) reducible fraction (F2), (3) oxidizable fraction (F3), and (4) residual fraction (F4). Various factions of As were extracted using BCR sequential extraction on soil samples collected on Days 1, 10, 20, 30, 50, 70 and 100, the results are shown in Figure S4a–g. As can be seen, the As concentrations of the four bound As in the soil were in the order of F4 > F2 > F3 > F1 with different proportions of Fe_3_O_4_@MBC at different incubation times. Compared with CK, for the MBC treatments, the F4 concentration decreased by 0.27–17.14% and the F1, F2, and F3 concentrations increased by 0.07–1.80%, 0.85–14.14%, and 0.10–3.83%, respectively. For Fe_3_O_4_@MBC treatments, F1 concentrations in the soils decreased more obviously than that of MBC. To be specific, the F4 concentration increased by 0.74–30.34% and the F1, F2, and F3 concentrations decreased by 0.04–1.78%, 0.04–28.57%, and 0.49–3.87%, respectively, which was attributed to the transformation of As from non-specific adsorbed and/or specifically adsorbed states to crystalline Fe/Al oxides and/or residual states in mining soils facilitated by iron-based biochar [16,17]. Moreover, it was also due to magnetic biochar’s capability of reducing the bioavailability and mobility of As through As complexation with O-containing functional groups in the magnetic biochar [19]. As can be clearly seen in Figure S4, the content in F4 fraction As was 94.24% on the 100 th incubation day. This illustrates that Fe_3_O_4_@MBC was more effective in facilitating the transformation of soil labile As to soil residual As than that MBC.

#### 3.2.4. TCLP Arsenic

**Figure 4:**
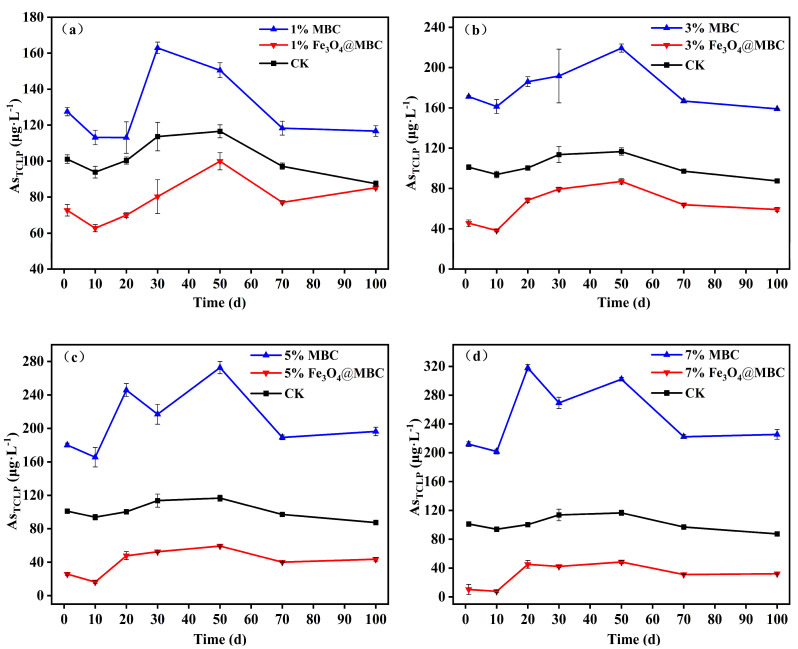
(**a**–**d**). As_TCLP_ content change of 1%, 3%, 5%, 7% MBC and Fe_3_O_4_@MBC amended soil in 1–100 d.

Usually, TCLP tests are used to evaluate the dissolution and mobility of As [27,46]. The TCLP extractable As concentrations indicated that the addition of Fe_3_O_4_@MBC at 1%, 3%, 5% and 7% had significant differences at incubation days 1 to 100 compared with those in the control group (*p* < 0.05), and it was dosage-dependent for As as the addition of Fe_3_O_4_@MBC (Figure 4). Specifically, the addition of the 7% Fe_3_O_4_@MBC treatment had a lower concentration of As_TCLP_ than that of the control; in addition, the concentration of As_TCLP_ was lowest at incubation day 10, which was lower than the international standards for drinking water quality (WHO) of 10 μg·L^−1^, while MBC addition elevated the As_TCLP_ concentration. The addition of 7% Fe_3_O_4_@MBC could effectively reduce the concentration of TCLP_As_ by 75% at incubation day 100 (Figure 4d). Interestingly, the addition of Fe_3_O_4_@MBC at 5% and 7% showed the same effect on the reduction in TCLP arsenic concentration. This can be explained by reaching adsorption saturation between a certain amount of material and soil available arsenic. Figure 4a shows that the soil aging time effect could slightly reduce the leaching risk of As (As_TCLP_ concentration in CK was lowest at incubation day 100). Biochar and oyster shell composites decreased soil As_TCLP_ concentration by 48.9% [25]. Based on the TCLP analysis, using Fe_3_O_4_@MBC to remediate As-contaminated soil was confirmed to be feasible. The results showed that Fe_3_O_4_@MBC was more effective than MBC in stabilizing As.

## 4. Discussion

### 4.1. Correlation Analysis of Soil Parameters

**Figure 5:**
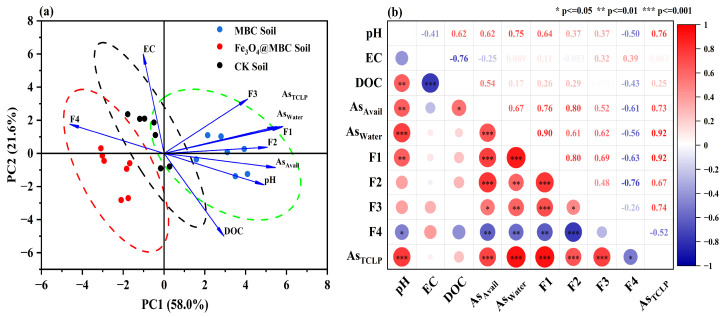
(**a**). Principal component analysis (PCA) of different response variables; (**b**). Pearson’s correlation of different response variables. Red and blue discs represent positive and negative relationships, respectively.

To better evaluate the influence of CK, MBC and Fe_3_O_4_@MBC treatment changes in soil parameters, a principal component analysis (PCA) was conducted [33]. The first and second principal components accounted for 58.0% and 21.6% of the total variables (Figure 5a), respectively, indicating that the presence of the soil passivator resulted in the difference. Specifically, pH, As_avail_, As_water_, As_TCLP_, F4, F2 and F1 were the elements that predominantly contributed to PC1. In contrast, PC2 was dominated by F3, DOC and EC, which were moderately correlated with PC2. These results illustrate that pH, DOC, and EC had moderate effects on As speciation (As_avail_, As_water_, As_TCLP_, F1, F2, F3 and F4). The locations of the passivator treatments varied and were grouped in the regions where treatments with similar effects on As speciation were grouped (Figure 5a). These were CK soil, MBC soil treatment and Fe_3_O_4_@MBC soil treatment. The overlapping section of the PCA indicates that the CK soil shares similarities with the MBC and Fe_3_O_4_@MBC soils in terms of collaborative treatment effectiveness (e.g., in EC and DOC). In conclusion, the efficacy of biochar treatment is dependent on the effective states of As in contaminated soil. 

The Pearson correlation coefficient measures whether two datasets are on the same line and is used to measure the linear relationship between variables [13,15]. To investigate the effects of Fe_3_O_4_@MBC on soil physicochemical properties and on the As fraction, a Pearson correlation matrix (Figure 5b) was performed on the soil pH, EC, DOC, As_avail_, As_water_, F1, F2, F3, F4 and As_TCLP_. Soil pH can affect the metal species in soil by changing the adsorption position and coordination properties of heavy metals. pH was significantly and positively correlated with As_avail_, As_water_ and As_TCLP_ (*p* < 0.001), indicating that the availability of As increased when soil pH increased (Figure 5b). This is because OH^−^ can compete with As for complexation sites with Fe(Ⅲ) mineral surfaces under high pH conditions, making anionic dissociated and released into the soil [32]. This is also the main reason that mobile As transforms to residual state after Fe_3_O_4_@MBC treatment in soil. In addition, there was a significantly positive correlation between As_avail_ and DOC in Fe_3_O_4_@MBC treatment soil (*p* < 0.05), which is similar to previous studies indicating that high concentrations of soil DOC can enhance the availability of heavy metals [27]. Overall, the addition of Fe_3_O_4_@MBC lowered soil EC within 100 days, immobilized soil DOC, slowed down the rate of its decomposition, and reduced bioavailable arsenic in the soil. This allowed the adsorption and pH regulation qualities of Fe_3_O_4_@MBC to utilize their ability to immobilize As in the soil fully.

### 4.2. Immobilization Mechanisms of Fe_3_O_4_@MBC

#### 4.2.1. XRD Analysis

**Figure 6:**
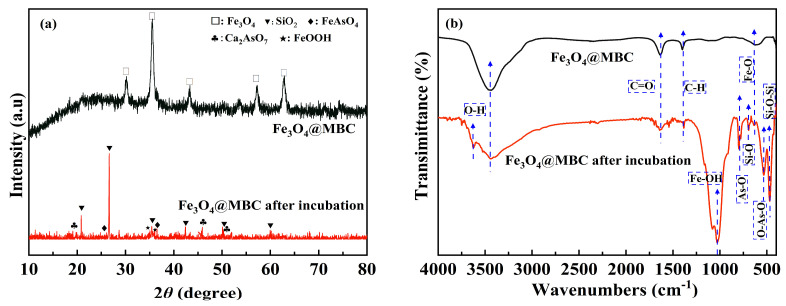
XRD and FTIR spectra of pristine Fe_3_O_4_@MBC and Fe_3_O_4_@MBC after 100 d of incubation: (**a**) XRD spectra and (**b**) FTIR spectra.

According to Figure 6a, the dominant phases in the Fe_3_O_4_@MBC were Fe_3_O_4_ (2θ = 30.09°, 35.43°, 43.07°, 53.44°, 56.94° and 62.53°), revealing that well-crystallized Fe_3_O_4_ was successfully synthesized and embedded in biochar. The main peaks in the Fe_3_O_4_@MBC after incubation indicated that there were mainly SiO_2_ and some amorphous, poorly crystallized precipitates (Ca_2_As_2_O_7_, FeO(OH) and FeAsO_4_). The weakening of the typical peaks of Fe_3_O_4_ indicates the incomplete separation of Fe_3_O_4_@MBC composites from the soil, while the formation of insoluble new minerals of FeAsO_4_ (2θ = 26.36°, 37.6°) was favorable for As immobilization. Ca_2_As_2_O_7_ was identified at 2θ of 19.07°, 46.03° and 51.91°, which could participate in As passivation with the generation of Ca-As coprecipitates. This implies that the released Ca species in soil that have not been completely separated could be consumed. However, the Ca-As complex is unstable and tends to partially release As partially into the soil [27]. The weak peaks of low crystallinity FeO(OH) could were observed at 2θ of 35.10° and 36.06°. The FeO(OH) in the Fe_3_O_4_@MBC could provide abundant active sites for the adsorption/complexation/coprecipitation of unstable As on its surface and lattice, contributing to the generation of Fe-As-O complexes/coprecipitates [23,47]. It can be concluded that FeO(OH) in Fe_3_O_4_@MBC plays a critical role in As passivation in soil.

#### 4.2.2. FTIR Analysis

The FTIR characteristic bands for -OH appeared at the wave number (ν) values of 3442 cm^−1^ and 3630 cm^−1^; for C=O at ν = 1638 cm^−1^, for C-H at ν = 1392 cm^−1^; for Fe-OH at ν = 1023 cm^−1^; for As-O at ν = 802 cm^−1^; for Si-O at ν = 692 cm^−1^; and for Si-O-Si at ν = 426 cm^−1^ (Figure 6b). This demonstrates that the peaks of the -OH and C=O groups decreased and shifted, indicating that the apparent oxygenic functional groups were related to the complexation of As because the O in O-H and C=O groups can form complexes with As by electron pair donation [48]. The appearance of O-As-O stretching at ν = 527 cm^−1^ suggests that As(III) was attached to the Fe oxides through complexation. In addition, the Fe-O band at ν = 630 cm^−1^ implied that As(III) adsorption could change the oxidizing species [49]. Meanwhile, Fe-OH was observed after incubation, which can cause ligand exchange between As and -OH to generate Fe-HAsO_4_^2−^/Fe-AsO_4_^3−^ [50]. According to these changes in the surface functional groups of Fe_3_O_4_@MBC before and after incubation, the surface adsorption caused by complexation and electrostatic interaction complexation may be responsible for the Fe_3_O_4_@MBC immobilization of As in soil.

#### 4.2.3. XPS Analysis

**Figure 7:**
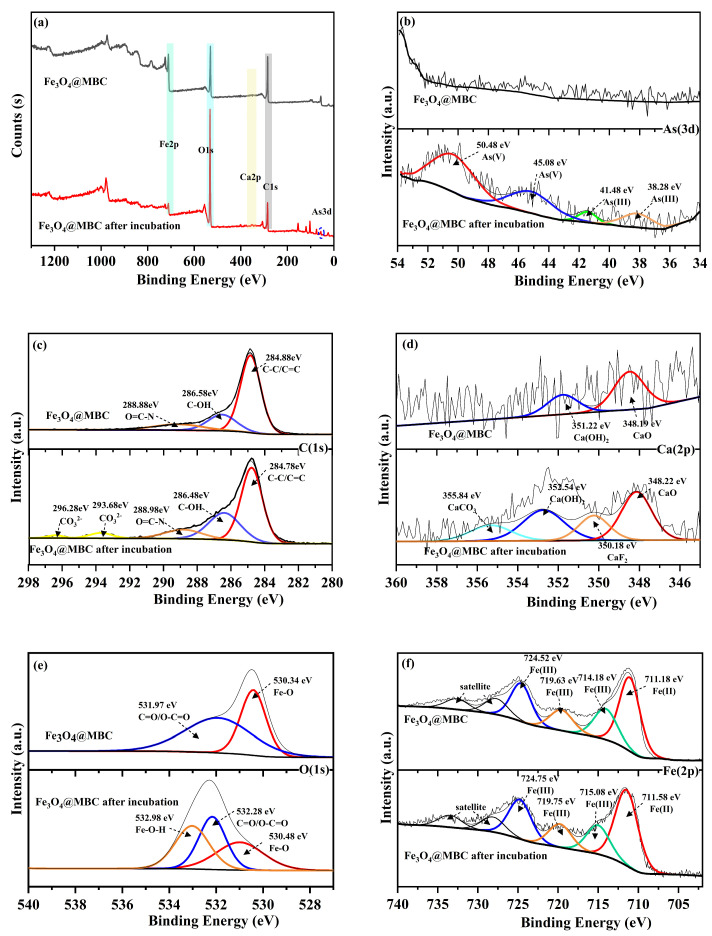
XPS spectra of pristine Fe_3_O_4_@MBC and Fe_3_O_4_@MBC after 100 d of incubation: (**a**) XPS wide-scan spectra, sub-peak fitted spectra of As 3d (**b**), C 1s (**c**), Ca 2p (**d**), O 1s (**e**) and Fe 2p (**f**).

From the obtained XPS wide-scan spectrum, it can be observed that C, O, Fe, Ca exist in Fe_3_O_4_@MBC before and after incubation, and the photoelectron peak of As 3d appears, which is attributed to the immobilization of As onto the Fe_3_O_4_@MBC surface (Figure 7a). The As 3d region was deconvoluted into four different peaks, where As(V)-O corresponds to 50.48 eV and 45.08 eV, and As(III) corresponds to 41.48 eV and 38.28 eV (Figure 7b). The peak positions in the C 1s spectrum before and after incubation were changed insignificantly, and the peaks at 284.88 eV, 286.58 eV and 288.88 eV were correlated with C-H, C-OH, and O=C-N [31], respectively (Figure 7c). The C=C/C-C groups decreased remarkably after incubation, whereas the oxygen-containing functional groups of C-OH and O=C-N increased. The lone-pair electrons in the oxygen-containing functional groups can combine with the empty arsenic orbitals to undergo coordination and complexation reactions [25,51]. Therefore, the increase in surface oxygen-containing functional groups was favorable for the immobilization of toxic trace elements [52], in accordance with the FTIR analysis. Meanwhile, in the O 1s spectrum, the C=O/O-C=O peak shifted to a lower binding energy, and the peak ratio of the metal oxides (Fe-O and Fe-OH) increased after the reaction (Figure 7e). This change occurs because while Fe-O participates in As oxidation, Fe-O adsorbs As through complexation and carboxyl groups can also participate in the complexation of As [45]. There were two characteristic peaks of Ca (348.19, 351.22 eV) before incubation, which intensively increased after incubation, indicating that ion exchange occurred during incubation (Figure 7d). Owing to the abundant calcium content in the soil, two new peaks appeared for the recycled Fe_3_O_4_@MBC (350.18 eV and 355.84 eV) [27]. For Fe, the binding energy position of the Fe 2p spectrum did not change significantly before and after incubation; Fe(III) (Fe 2p peaks at 724.75 eV) was dominant, and the content of Fe(II) (Fe 2p peaks at 711.58 eV) in Fe_3_O_4_@MBC decreased after incubation, indicating that Fe(II) was partially oxidized into Fe(III) in the incubation process (Figure 7f).

#### 4.2.4. Mechanism Analysis

**Figure 8:**
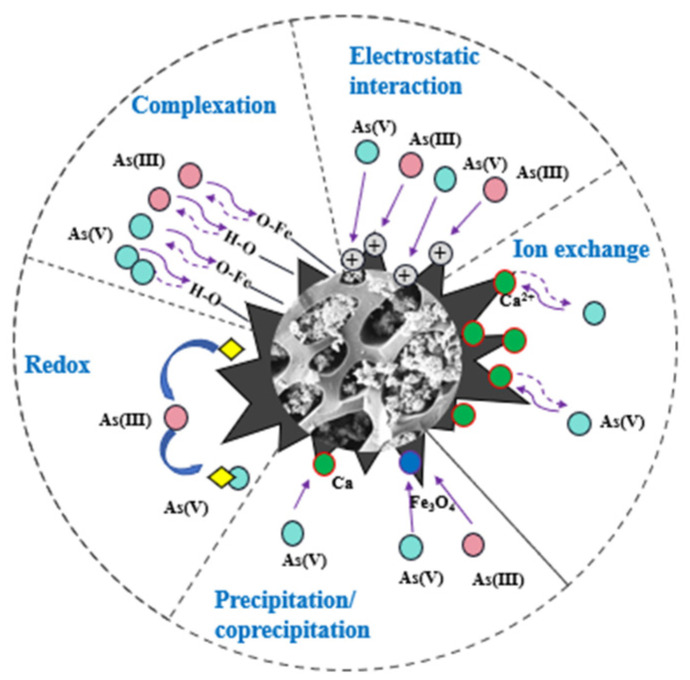
The proposed mechanism of Fe_3_O_4_@MBC remediation for arsenic-contaminated soil.

Based on the above analysis, the proposed remediation mechanism of As immobilization in the soil by Fe_3_O_4_@MBC involves redox reactions, complexation, electrostatic attraction, surface adsorption, and coprecipitation. Following the addition of Fe_3_O_4_@MBC into the soil, (1) firstly, as the pH and EC values increased, electrostatic adsorption and ion exchange took place to replace Si-OH/Fe-OH/O-H and other groups of alumino-silicates and Fe-oxo-hydroxides on its surface, which were widely adsorbed and enriched on the material’s surface. (2) Secondly, depending on the results of the XPS analyses, it was evident that during the incubation process, Fe(III) was reduced to Fe (II), and As(III) was oxidized by Fe to the more inert As(V). This suggests that co-precipitation or surface complexation occurred to form Fe-As complexes, or that As was immobilized to form insoluble minerals. (3) Lastly, the XRD analysis results revealed that the changed materials’ secondary mineralization formed crystalline hydroxyl iron oxides, which in turn stimulated the development of more stable multiphase minerals to achieve the sequestration of pollutants containing arsenic. The possible mechanism of Fe_3_O_4_@MBC remediation for arsenic-contaminated soil is illustrated in Figure 8.

## 5. Conclusions

Fe_3_O_4_@MBC slightly increased the soil pH and soil DOC concentration, and the magnitude of the increase was lower than that of MBC. Fe_3_O_4_@MBC significantly improved the immobilization efficiency of biochar towards As in contaminated soils, and the immobilization efficiency was positively correlated with the addition of Fe_3_O_4_@MBC dosage and incubation time. Fe_3_O_4_@MBC treatment efficiently converted the active species of As to residual fractions, reducing its bioavailability. The addition of Fe_3_O_4_@MBC decreased the concentrations of bioavailable As. The As_avail_ content in the Fe_3_O_4_@MBC 7% treatments was the lowest (64.58%) which was only 9.08% and 3.39% lower than that of the Fe_3_O_4_@MBC 3% and Fe_3_O_4_@MBC 5% treatments, respectively. Therefore, from an economic perspective, we recommended using 3% or 5% of the addition ratio in large-scale As-contaminated soil remediation. The BCR extraction further confirmed that Fe_3_O_4_@MBC could transform the unstable As faction into a stable fraction. Fe_3_O_4_@MBC could remediate As mainly through (1) redox reactions; (2) the formation of the surface complexes on the surface of Fe oxide in Fe_3_O_4_@MBC; and (3) the great adsorption ability of the porous Fe_3_O_4_@MBC surface. It appears that Fe_3_O_4_@MBC is an efficient and environmentally friendly amendment to remediate As contamination in soil.

## Data Availability

The data presented in this study are available on request from the corresponding author.

## Supplementary Materials

The following supporting information can be found online at https://www.mdpi.com/article/10.3390/toxics12110765/s1, Table S1: Basic physical and chemical properties of the soil; Table S2: Arsenic form in the test soil; Table S3: The element analysis of element MBC and Fe_3_O_4_@MBC; Figure S1. (a–d): The effects on the soil pH value after adding 1–7% MBC and Fe_3_O_4_@MBC; Figure S2. (a–d): Dynamic change in the soil EC value after adding 1–7% MBC and Fe_3_O_4_@MBC; Figure S3. (a–d): Dynamic change in the soil EC value after adding 1–7% MBC and Fe_3_O_4_@MBC; Figure S4 (a–g): The speciation fraction of As in the soil after applying 1–7% MBC Fe_3_O_4_@MBC; Figure S5.: The relationship between the Zeta potential and pH for MBC and Fe_3_O_4_@MBC.
