# Supplementary material for: Fe3O4/Mulberry Stem Biochar as a Potential Amendment for Highly Arsenic-Contaminated Paddy Soil Remediation
Source: Toxics. 2024 Oct 22;12(11):765. doi: 10.3390/toxics12110765 (PMC11598391; doi:10.3390/toxics12110765)
Supplement: Supplementary file 1 [file toxics-12-00765-s001.zip › toxics-3258513-supplementary.pdf]

Supplementary Materials

Fe_3O_4/Mulberry stem biochar as a Potential Amendment for Highly Arsenic-Contaminated Paddy Soil Remediation

Ziling Tang[1], Meina Liang[1,2,3*], Yanmei Ding[1], Chongmin Liu[1,2], Qing Zhang[1,2], Dunqiu Wang[1,2,3], Xuehong Zhang[1,2,3]

[1] College of Environmental Science and Engineering, Guilin University of Technology, Guilin 541004, China

[2] Guangxi Key Laboratory of Environmental Pollution Control Theory and Technology, Guilin University of Technology, Guilin 541006, China

[3] Guangxi Collaborative Innovation Center for Water Pollution Control and Water Safety in Karst Area, Guilin University of Technology, Guilin 541006, China

* Correspondence: liangmeina@glut.edu.cn

Content

Table S1. Basic physical and chemical properties of the soil.

Table S2. Arsenic form in the test soil.

Table S3. The element analysis of element MBC and Fe_3O_4@MBC.

Figure S1 (a–d).: The effects on the soil pH value after adding 1 % – 7 % MBC and Fe_3O_4@MBC.

Figure S2 (a–d).: Dynamic change in the soil EC value after adding 1 % – 7 % MBC and Fe_3O_4@MBC.

Figure S3 (a–d).: Dynamic change in the soil EC value after adding 1 % – 7 % MBC and Fe_3O_4@MBC.

Figure S4 (a–g): The speciation fraction of As in the soil after applying 1% – 7% MBC and Fe_3O_4@MBC.

Figure S5.: The relationship between the Zeta potential and pH for MBC and Fe_3O_4@MBC.

Experiment reagents

Chloride hexahydrate(($FeCl_3 \cdot 6H_2O$), ammonium ferrous sulfate hexahydrate(($(NH_4)_2Fe(SO_4)_2 \cdot 6H_2O$), ferrous sulfate heptahydrate($FeSO_4 \cdot 7H_2O$), sodium bicarbonate($NaHCO_3$), sodium hydroxide($NaOH$), glacial acetic acid(CH_3COOH), ammonia($NH_3 \cdot H_2O$), hydrogen nitrate(HNO_3), and hydrochloric acid(HCl) used in this experiment were analytical grade reagents and purchased from Xilong Scientific Co. Ltd. (China).

The concentration of total As was 237.68 $mg \cdot kg^{-1}$(determined by atomic fluorescence spectrophotometer, AFS, Beijing Jitian Instrument Company SA-20, China) which was 1.58 folds higher than the soil risk control value (As 150 mg kg^{-1}, GB 15618-2018, $5.5 \leq pH < 6.5$).

Table S1 Basic physical and chemical properties of the soil.

Samples	pH	EC（us/cm）	DOC（mg/kg）	moisture content（%）	As (mg/kg）	Cu (mg/kg）	Pb (mg/kg）	Ni (mg/kg）	Zn (mg/kg）
Test soil	6.18	126.47	291.66	37.46	237.68	100.23	343.73	71.45	891.87

Table S2 Arsenic form in the test soil.

Samples	As_{Avail}（mg/kg）	As_{Water}（mg/kg）	F1（mg/kg）	F2（mg/kg）	F3（mg/kg）	F4（mg/kg）
Test soil	8.90	0.43	4.85	79.71	1.73	151.39

Table S3 The element analysis of element MBC and Fe_3O_4@MBC.

Materials	C（%）	H（%）	N（%）	S（%）
MBC	74.53	1.25	0.66	0.60
Fe_3O_4@MBC	25.41	0.83	0.39	0.37

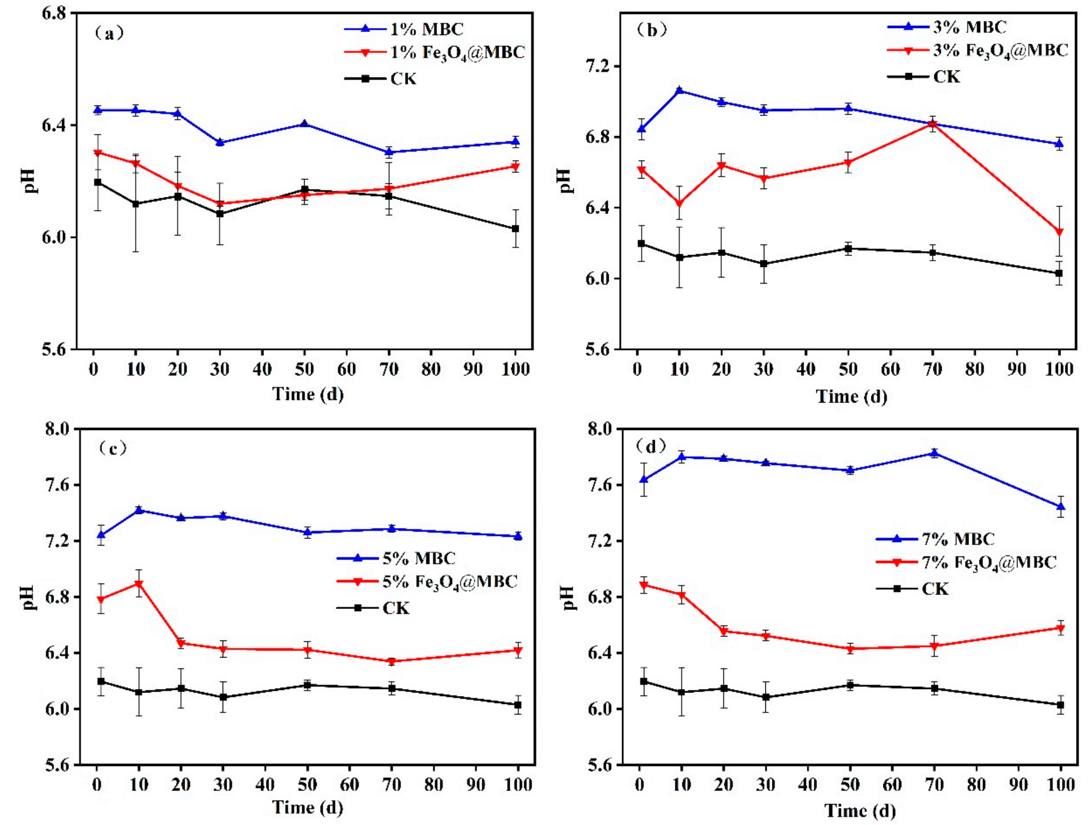

Figure S1. (a–d): The effects on the soil pH value after adding 1 % — 7 % MBC and Fe_3O_4@MBC.

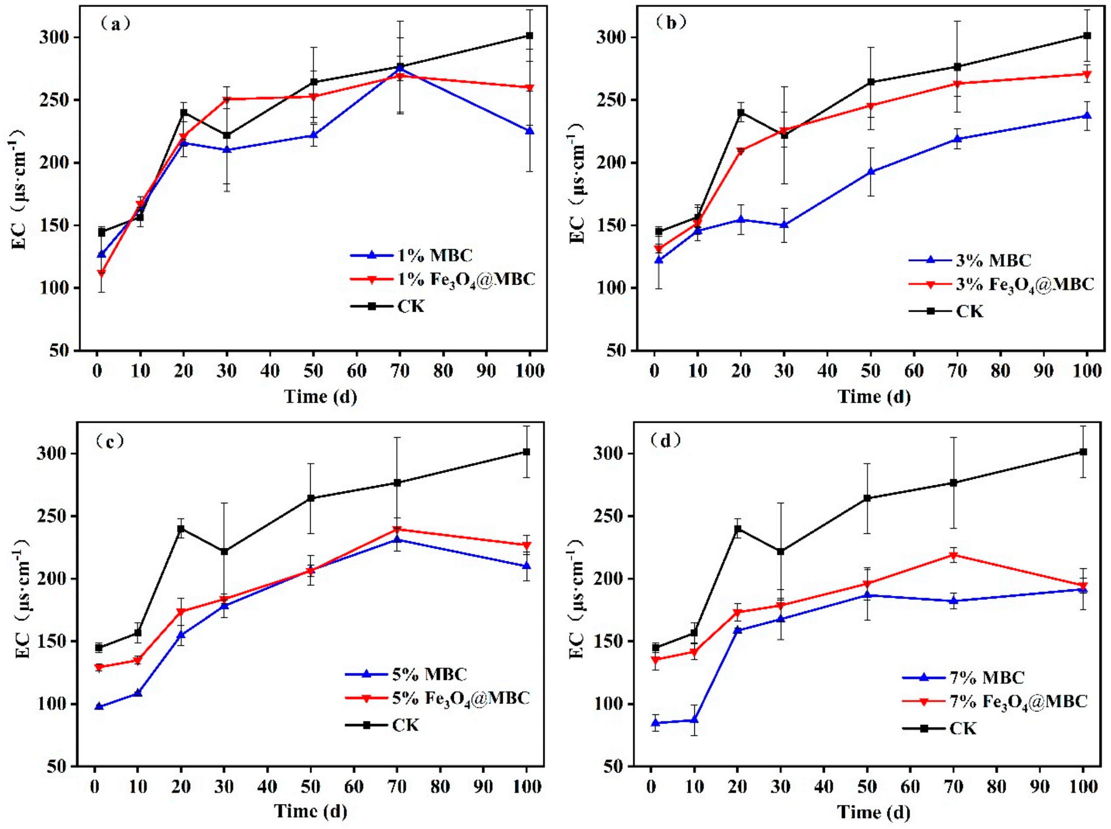

Figure S2. (a–d): Dynamic change in the soil EC value after adding 1 % — 7 % MBC and Fe_3O_4@MBC.

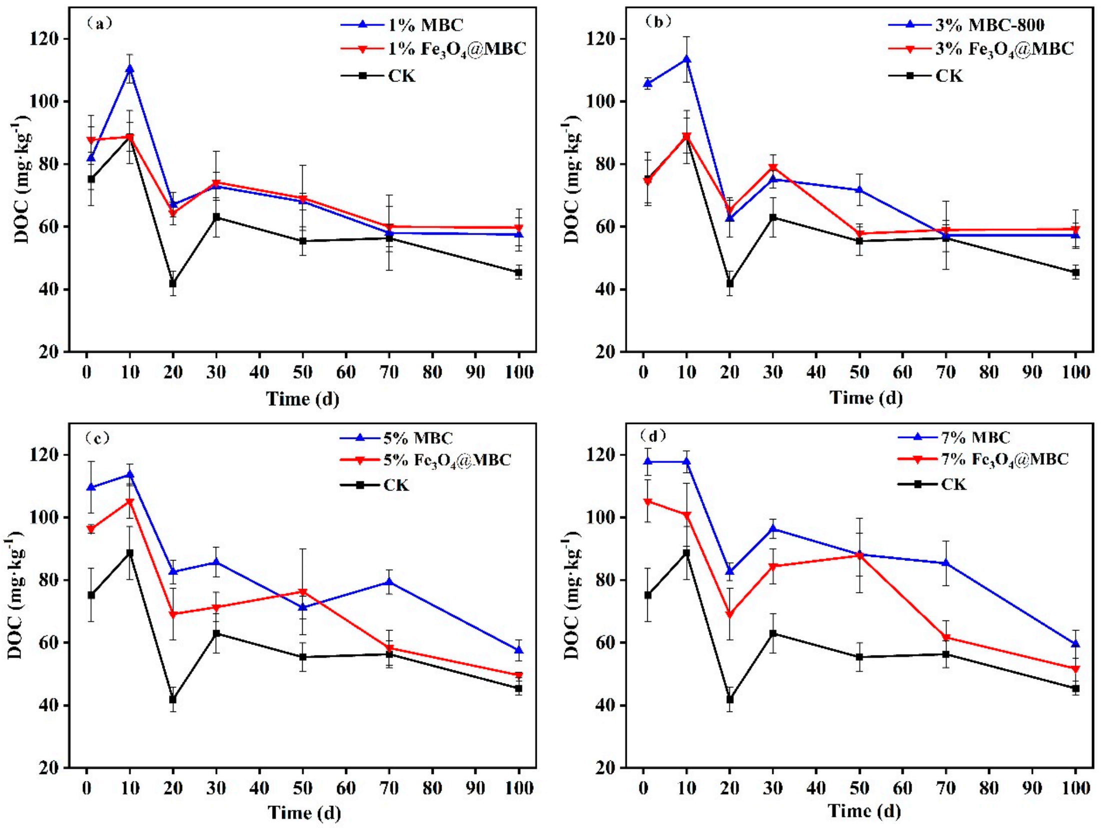

Figure S3. (a–d): Dynamic change in the soil EC value after adding 1 % — 7 % MBC and Fe_3O_4@MBC.

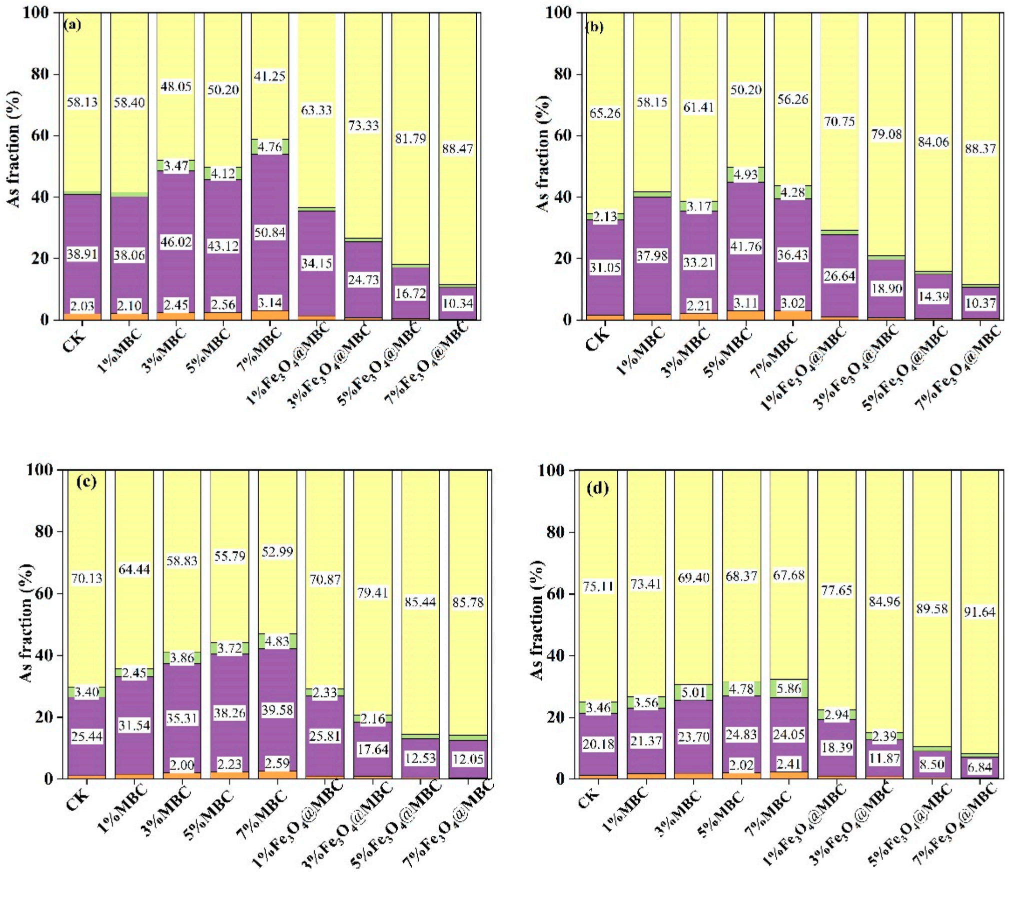

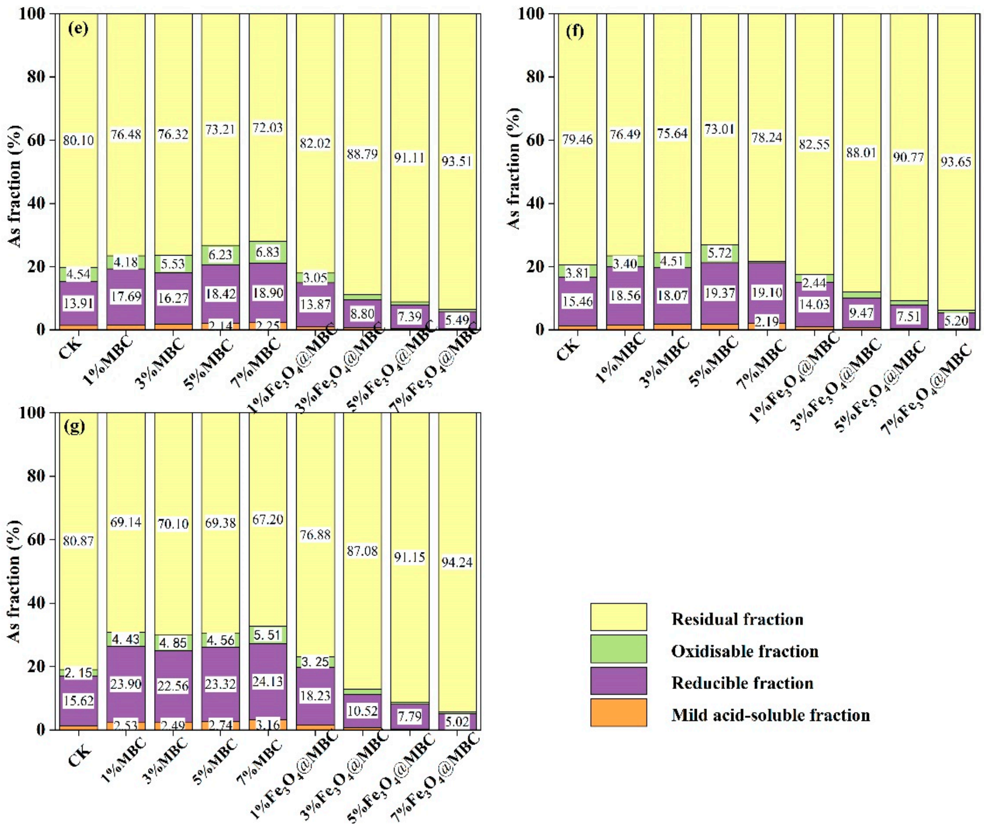

Figure S4. (a-g): The speciation fraction of As inthe soil after applying 1%-7% MBC and Fe_3O_4@MBC.

(a-g) soil sample was incubated 1 d, 10 d, 20 d, 30 d, 50 d, 70 d and 100 d, respectively).

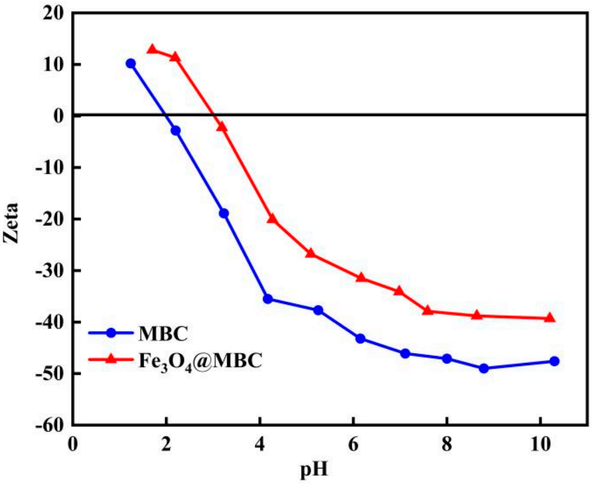

Figure S5. The relationship between Zeta potential and pH of MBC and Fe_3O_4@MBC.
